# Robust Activity Recognition via Redundancy-Aware CNNs and Novel Pooling for Noisy Mobile Sensor Data

**DOI:** 10.3390/s26020710

**Published:** 2026-01-21

**Authors:** Bnar Azad Hamad Ameen, Sadegh Abdollah Aminifar

**Affiliations:** 1 Computer Science Department, Faculty of Science, Soran University, Soran 44008, Erbil, Kurdistan Region, Iraq; 2 Mechatronic and Robotics Engineering Department, Faculty of Engineering, Soran University, Soran 44008, Erbil, Kurdistan Region, Iraq

**Keywords:** accelerometer signals, convolutional neural networks (CNN), human activity recognition, mobile sensors, noise robustness, pooling mechanisms, time-series to image transformation

## Abstract

This paper proposes a robust convolutional neural network (CNN) architecture for human activity recognition (HAR) using smartphone accelerometer data, evaluated on the WISDM dataset. We introduce two novel pooling mechanisms—Pooling A (Extrema Contrast Pooling (ECP)) and Pooling B (Center Minus Variation (CMV))—that enhance feature discrimination and noise robustness. ECP emphasizes sharp signal transitions through a nonlinear penalty based on the squared range between extrema, while CMV Pooling penalizes local variability by subtracting the standard deviation, improving resilience to noise. Input data are normalized to the [0, 1] range to ensure bounded and interpretable pooled outputs. The proposed framework is evaluated in two separate configurations: (1) a 1D CNN applied to raw tri-axial sensor streams with the proposed pooling layers, and (2) a histogram-based image encoding pipeline that transforms segment-level sensor redundancy into RGB representations for a 2D CNN with fully connected layers. Ablation studies show that histogram encoding provides the largest improvement, while the combination of ECP and CMV further enhances classification performance. Across six activity classes, the 2D CNN system achieves up to 96.84% weighted classification accuracy, outperforming baseline models and traditional average pooling. Under Gaussian, salt-and-pepper, and mixed noise conditions, the proposed pooling layers consistently reduce performance degradation, demonstrating improved stability in real-world sensing environments. These results highlight the benefits of redundancy-aware pooling and histogram-based representations for accurate and robust mobile HAR systems.

## 1. Introduction

Human Activity Recognition (HAR) has attracted significant attention in recent years due to the widespread adoption of smartphones and wearable devices equipped with inertial sensors. These embedded accelerometers continuously generate rich time-series data that enable the recognition of human activities, supporting a wide range of applications such as health monitoring, elder care, fitness tracking, and human–computer interaction [1,2].

Traditional HAR approaches have largely relied on handcrafted statistical features or conventional deep learning models, particularly convolutional neural networks (CNNs). However, real-world deployment of HAR systems remains challenging due to sensor noise, device orientation variability, and inter-user differences, all of which can substantially degrade classification performance [3,4]. To address these issues, recent studies have explored hybrid deep learning models, time–frequency representations, and attention-based mechanisms to improve feature robustness and discriminative power [5,6,7]. Despite these advances, most CNN-based HAR frameworks still rely on standard pooling operations, such as max or average pooling, which do not explicitly model uncertainty, noise, or signal variability. Moreover, conventional processing of raw sensor data often overlooks the statistical redundancy inherent in high-frequency and multi-axis measurements [8,9].

In this work, we focus on accelerometer-based HAR while maintaining a sensor-agnostic design philosophy. We propose a robust and redundancy-aware HAR framework that introduces two novel pooling operations—Pooling A and Pooling B—designed to capture meaningful statistical characteristics of sensor signals. Pooling A emphasizes extrema-based contrast to preserve transient motion patterns, while Pooling B incorporates variance-aware aggregation to suppress noise and reduce the influence of local fluctuations. These pooling mechanisms enhance feature robustness while remaining computationally lightweight, making them suitable for deployment on edge and embedded devices operating at typical HAR sampling rates (50–100 Hz).

To further enrich the learned representations, the proposed framework integrates histogram-based encoding and window-level statistical descriptors. This strategy enables a redundancy-aware representation of sensor data, capturing distributional characteristics without incurring significant computational overhead. As a result, the proposed approach improves classification performance while remaining efficient enough for real-time applications.

The proposed pooling strategies are integrated into two CNN-based architectures: (1) a 1D CNN that operates directly on normalized raw tri-axial accelerometer signals, and (2) a 2D CNN that processes RGB images derived from histogram-based encodings of the sensor data. This histogram-to-image representation captures intra-window distributional characteristics across sensor axes, enabling effective spatial feature learning and improved robustness to noise through structured visual patterns. The proposed framework is evaluated using the publicly available WISDM dataset [1], which includes labeled accelerometer recordings for six common daily activities. Experimental results demonstrate that the proposed approach yields notable improvements in classification accuracy and robustness, particularly under synthetic noise conditions designed to mimic real-world signal variability.

The remainder of this paper is organized as follows. Section 2 reviews related work in sensor-based human activity recognition and pooling strategies. Section 3 introduces the dataset and provides preliminary analytical insights. Section 4 presents the proposed methodology, including the CNN architectures, pooling mechanisms, and evaluation metrics. Section 5 reports experimental results, including ablation studies and computational cost analysis under both clean and noisy scenarios. Finally, Section 6 concludes the paper and discusses future research directions, including cross-dataset generalization, multimodal sensor fusion (e.g., gyroscope and magnetometer), and deployment on resource-constrained edge devices.

## 2. Related Work

Recent advances in sensor-based HAR have increasingly emphasized robustness to environmental noise and signal variability, especially within the context of real-world IoT applications. Numerous studies have explored a range of deep architectures, including CNNs, hybrid CNN-RNN models, and attention-enhanced frameworks, aimed at improving classification accuracy under diverse conditions. For instance, Genc et al. [10] proposed a fine-tuned CNN–LSTM model with small kernels and average pooling to capture detailed temporal–spatial features and Q. Zhang et al. [11] proposed a lightweight wearable HAR system using 1D CNNs, demonstrating the practical advantages of streamlined architectures. Likewise, Liu et al. [12] introduced the SETransformer, which integrates squeeze-and-excitation modules with transformer-based temporal modeling and learnable pooling for enhanced generalization under signal noise.

A growing body of work has addressed the challenge of noisy or uncertain input by embedding attention [13], adaptive pooling [14], or embedding-based denoising techniques [15]. These approaches demonstrate improved resilience to real-world sensor drift and user variance. However, most rely on standard max or average pooling, which are not explicitly designed to handle statistical outliers or preserve local distributional information critical in noisy HAR scenarios.

Within the WISDM dataset domain specifically, Kobayashi et al. [16] introduced MarNASNets, a family of CNNs optimized for wearable sensor efficiency. Sharen et al. [6] proposed WISNet, a deep CNN trained directly on raw sensor signals, achieving promising accuracy. Similarly, Abdellatef et al. [7] explored multi-layer CNNs but did not incorporate pooling strategies tailored to uncertainty. While Seelwal and Srinivas [4], and Min et al. [3] compared traditional ML classifiers on WISDM, none addressed redundancy modeling or custom pooling. Importantly, none of these studies explicitly evaluated model robustness under synthetic noise injection.

Handling sensor noise remains a critical challenge in HAR, as accelerometer data often suffers from measurement uncertainty, environmental interference, and sampling inconsistencies. Various studies have attempted to mitigate the effects of noise particularly Gaussian, salt-and-pepper, and speckle noise using robust modeling and preprocessing techniques. For instance, Chu et al. [17] employed an adaptive wavelet denoising approach to filter Gaussian noise in activity datasets, showing significant gains in classification accuracy. In a related study, Ma, X., & Liu, S. [18] examined the impact of salt-and-pepper noise and applied median filtering prior to LSTM classification to enhance signal integrity. A hybrid autoencoder–CNN model was proposed by Han et al. [19] to extract denoised latent features from sensor data distorted by speckle and additive noise. Furthermore, robust pooling strategies have been explored to counteract outliers, such as in the work of Tang et al. [20], where noise-resilient feature selection improved activity segmentation performance. Notably, Negri et al. [21] integrated uncertainty modeling into the HAR pipeline, emphasizing the role of noisy label correction and dropout-based regularization in deep networks. Despite these efforts, few studies have directly modeled statistical variability within the pooling layer or used custom transformations to encode intra-window redundancy. Beyond max and average pooling, several alternative pooling strategies have been introduced to improve robustness and generalization. Stochastic pooling selects activations within a pooling region according to a multinomial distribution proportional to their magnitude, thereby introducing randomness that reduces overfitting and enhances noise tolerance in CNNs [22]. Fractional max-pooling, on the other hand, applies pooling with non-integer strides, preserving more fine-grained structural information and avoiding excessive signal smoothing [23]. While both methods have shown benefits in image recognition tasks, their reliance on randomness and irregular pooling regions makes them less directly interpretable and harder to constrain within bounded ranges. By contrast, our proposed pooling mechanisms are deterministic, normalization-compatible, and tailored to preserve transient temporal features in sensor data, making them more suitable for HAR under noisy conditions. Our method addresses this gap by introducing range- and deviation-sensitive pooling layers, explicitly tested under multiple synthetic noise types to evaluate stability and generalization.

Noise-aware modeling, as seen in recent works on uncertainty quantification [24,25,26], suggests that defuzzification and uncertainty avoidance strategies can play a critical role in signal segmentation and classification. Such ideas have been explored in fuzzy image segmentation [25,27,28] and recommender systems [29,30,31], often within the IoT ecosystem [32]. These studies highlight the importance of stability in data-driven systems operating in uncertain environments, a principle echoed in our custom pooling designs.

Pooling mechanisms and multi-scale feature aggregation play a critical role in improving the robustness and representational capacity of convolutional neural networks across diverse application domains. Xu et al. [24] proposed a multi-scale CNN combined with attention mechanisms for remaining useful life prediction using multi-sensor signals, demonstrating that hierarchical feature extraction and adaptive weighting can significantly enhance robustness to signal variability. Several studies have specifically examined the impact of pooling strategies on CNN performance; Zafar et al. [25] conducted a comprehensive comparison of common pooling methods, highlighting trade-offs between information preservation and computational efficiency. Beyond conventional max and average pooling, task-specific pooling layers have been introduced to capture richer statistical and structural information, such as the novel pooling layer proposed by Mohamed et al. [26] for thermographic breast cancer analysis. Attention-based pooling approaches have also shown effectiveness in handling complex spatial patterns, as demonstrated by Bi et al. [33] through multi-scale stacking attention pooling for remote sensing scene classification. Comprehensive surveys further emphasize that pooling design significantly influences model robustness, generalization, and sensitivity to noise, particularly in medical and signal-based applications [34]. While these works underscore the importance of pooling and attention mechanisms, most are designed for image-based or industrial signals and do not explicitly address redundancy-aware pooling for noisy mobile sensor time-series data, which is the focus of the present study.

Robust activity recognition under noisy sensing conditions has received increasing attention across multiple modalities, including audio-visual and wearable sensor data. Noise-tolerant learning strategies have been explored beyond inertial sensing; for example, Han et al. [35] proposed a noise-resilient framework for audio-visual action recognition that explicitly mitigates corrupted modalities, demonstrating the importance of redundancy-aware representations. Similarly, recent work on background-noise-aware modeling in speech processing highlights the benefits of explicitly accounting for noise characteristics during feature transformation and inference [36]. In the context of sensor-based HAR, Miah et al. [37] introduced a multi-stream architecture with time-varying features and attention-based dimensionality reduction to enhance robustness against sensor variability. Choudhury and Soni [38] addressed uncontrolled environments by combining CNNs and LSTMs with adaptive batch sizing to improve stability under noisy and non-stationary data. Comprehensive reviews further emphasize that noise, sensor displacement, and redundancy remain central challenges in HAR using smart devices, calling for architectures that can robustly aggregate information across time and feature dimensions [39]. While these approaches improve robustness through multimodal fusion, temporal modeling, or attention mechanisms, they largely rely on conventional pooling strategies. In contrast, our work focuses on redundancy-aware CNN representations and novel pooling mechanisms explicitly designed to stabilize feature aggregation under noisy mobile sensor conditions.

Our work is distinguished by the integration of redundancy-aware histogram-based encoding and statistically grounded pooling layers. Pooling A penalizes wide extrema ranges, while Pooling B subtracts intra-window standard deviation enhancing both feature contrast and noise suppression. The transformation of tri-axial accelerometer data into RGB histograms further enables the use of 2D CNNs to capture local spatial dependencies.

To the best of our knowledge, no previous study has combined statistical feature encoding with custom pooling mechanisms within a unified CNN framework for HAR, particularly not with explicit evaluation under various synthetic noise scenarios.

## 3. Dataset Description

This study uses the Wireless Sensor Data Mining (WISDM) dataset, introduced by Kwapisz et al. [1], which has become a foundational benchmark for mobile sensor-based human activity recognition. The dataset was collected as part of the WISDM Lab’s early work on activity classification using smartphone accelerometers. It captures raw time-series data from users performing physical activities while carrying a consumer-grade mobile phone.

### 3.1. Data Collection Methodology

The WISDM dataset was constructed using an Android-based smartphone equipped with a built-in 3-axis accelerometer. Participants were instructed to carry the device in their front pants pocket while performing a sequence of predefined physical activities. The phone continuously recorded x, y, and z acceleration values at a frequency of 20 Hz, which was considered sufficient for activity pattern recognition at the time. Six types of physical activities were targeted: Walking, Jogging, Sitting, Standing, Upsatirs (ascending staris), and Downstairs (descending stairs).

Data was collected from 36 subjects across multiple sessions, yielding over 1 million labeled accelerometer readings. Each reading in the dataset is a timestamped sample consisting of a user ID, activity label, and acceleration values along the three spatial axes.

The raw data was segmented into 10-s windows, equivalent to 200 samples per window, assuming a nominal 20 Hz sampling rate. Labels were assigned per window based on the majority activity performed within that period. Technical specifications are shown in Table 1; user-activity frequencies and cumulative statistics regarding data are shown in Table 2 and Table 3, respectively.

This study is limited by its reliance on the WISDM dataset, which lacks transitional complexity and may constrain generalization. Future validation on richer datasets (e.g., UCI-HAR, PAMAP2, MobiAct) is needed. Moreover, only accelerometer data were used, whereas incorporating multimodal signals such as gyroscopes and magnetometers could enhance robustness for subtle activities. Although Pooling A and B remain lightweight, their added computations (range and standard deviation) should be benchmarked for latency and energy efficiency on mobile and edge devices. Finally, extending the approach beyond HAR to domains like speech, ECG, and industrial IoT offers promising avenues for broader applicability.

### 3.2. Necessary Preprocessing for Human Activity Recognition (HAR) Models

The preprocessing of HAR datasets plays a critical role in ensuring reliable and generalizable model performance. Based on an in-depth analysis of the WISDM dataset, we identify and address three major challenges:

Class Imbalance: Observation: Activities such as Walking and Jogging are significantly overrepresented, while sitting and standing appear less frequently. This leads to a skewed distribution across class labels.

Implication: A classifier trained on such data may become biased towards majority classes, resulting in poor recognition of minority activities.

Applied Solution: Class weighting in the loss function (e.g., CrossEntropyLoss (weight = class_weights) in PyTorch 2.9.0) is set to penalize misclassification of minority classes more heavily.

User-Specific Behavior Variability: Observation: The execution of activities varies across users due to physiological differences, movement styles, and contextual factors.

Implication: Generic models trained on aggregate data may underperform on unseen users, particularly when activity dynamics differ.

Applied Solution: User-aware modeling is used, including user ID as a feature.

Dataset Strengths: Naturalistic usage: Subjects carried phones in a natural way (in their pockets), making data more reflective of real-world use compared to sensor attachments. Simplicity and scale: The dataset’s straightforward design and scale make it highly accessible for baseline and deep learning studies. Broad influence: It has been widely cited and used in both traditional machine learning and deep learning HAR research [1].

Limitations and Challenges: Despite its historical importance, the WISDM dataset has several critical limitations that modern HAR systems must address:

Sampling Inconsistency: Although the nominal sampling rate is 20 Hz, the actual rate fluctuates due to background processes on the smartphone OS. This leads to irregular time intervals between samples, violating assumptions of uniform sampling often required by deep learning models.

Missing Data: Some windows contain incomplete samples or abnormal interruptions due to phone behavior (e.g., temporary suspension, buffering delays). These missing segments are not explicitly annotated, which can lead to misleading training patterns, inaccurate temporal modeling, and noise amplification in CNN filters.

Windowing Strategy: While the WISDM dataset originally employs non-overlapping 10s windows—potentially missing activity transitions and reducing temporal continuity—we additionally evaluated overlapping windows with a 2 s (20%) overlap, similar to strategies used in PAMAP2 and UCI-HAR [40], to enrich training samples and better capture transitional dynamics.

Fixed Device Position: All participants carried the phone in the same front pocket location. This eliminates variability in device orientation or placement (e.g., backpack, armband), limiting the dataset’s generalizability to other phone-carrying configurations.

Lack of Gyroscopic Data: Only accelerometer readings are recorded. No gyroscope, magnetometer, or GPS data are included. This restricts feature richness and sensor fusion opportunities.

Labeling Bias: Labels are assigned per window based on majority rule, which can misclassify transitional windows where users switch from one activity to another.

Demographic Homogeneity: Although 36 users participated, demographic information (e.g., age, gender, fitness level) was not published. This raises concerns about user diversity and generalization across populations.

### 3.3. Relevance to This Study

**Figure 1:**
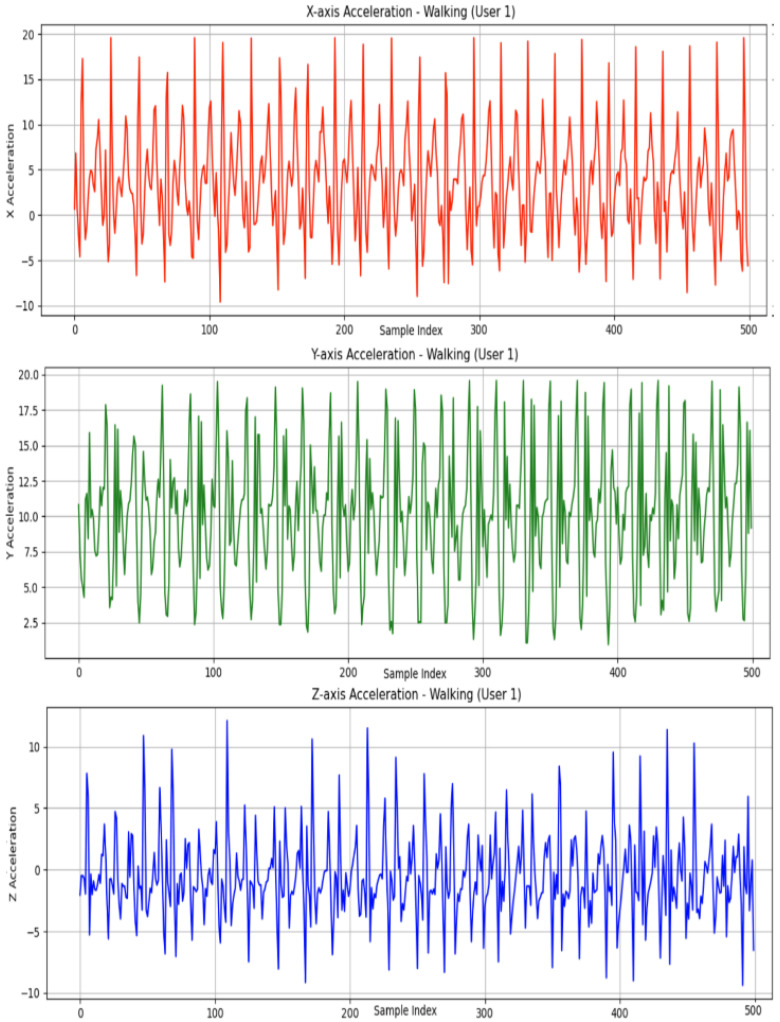
Raw accelerometer signal curves along the X, Y, and Z axes.

Despite its limitations, the WISDM dataset is ideally suited to evaluating redundancy-aware pooling and histogram-to-image encoding methods. Each activity is sampled with high temporal redundancy due to the 20 Hz rate and continuous motion. This allows effective aggregation of intra-window patterns and facilitates the histogram-based RGB image construction used in our proposed 2DCNN path. As illustrated in Figure 1, the raw accelerometer readings along the X, Y, and Z axes exhibit distinct temporal patterns corresponding to different physical movements.

Furthermore, the dataset’s lack of noise annotation makes it an ideal candidate for synthetic noise injection experiments, as performed in this work (Gaussian, salt-and-pepper, and mixed noise). This allows us to benchmark the noise robustness of traditional vs. proposed pooling strategies under controlled yet realistic sensor uncertainty.

## 4. Methodology

**Figure 2:**
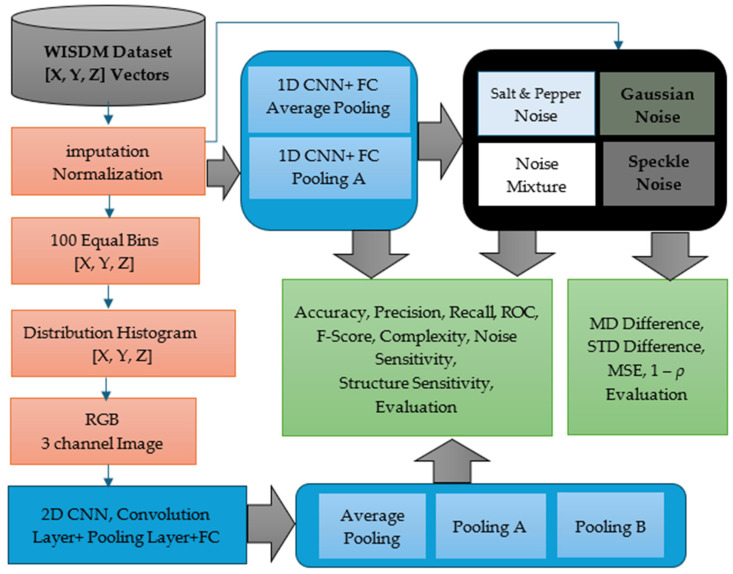
Overview of the proposed HAR methodology.

Our proposed system comprises two parallel processing branches: one based on raw time-series accelerometer data and the other on histogram-based RGB image representations. Both employ enhanced CNN architectures equipped with specialized pooling mechanisms to improve robustness and discrimination in human activity recognition (HAR). Figure 2 illustrates the overall methodology, including preprocessing, classification, and noise analysis components.

### 4.1. Pooling Mechanisms

To replace conventional max or average pooling, we introduce two novel pooling strategies:

Pooling A for array X is defined as

$$\mathrm{Pooled}(X)=\;\frac{max(X)+min(X)}{2}-\frac{{\left(max\left(X\right)-min(X)\right)}^{2}}{2}$$

Pooling B for array X is defined as

$$\mathrm{Pooled}(X)=\frac{max(X)+min(X)}{2}-std(X)$$

Pooling A computes the average of extrema and penalizes the range nonlinearly, emphasizing sharp transitions and making it ideal for dynamic activities.

Pooling B subtracts the standard deviation from the extrema average, promoting noise resilience through more stable aggregation.

Pooling A (Extrema Contrast Pooling (ECP)) and Pooling B (Center Minus Variation (CMV)) are named to reflect the core operations they perform. ECP captures the midpoint between the maximum and minimum values while penalizing large spreads via the squared range, emphasizing contrast between extrema. CMV, on the other hand, centers on the average of the extreme values and adjusts it by the standard deviation, effectively accounting for the overall variation in the data. Together, these names provide intuitive insight into the functional behavior of each pooling method.

There is an important prerequisite for applying both proposed pooling mechanisms: the input data must be normalized to the [0, 1] range. This normalization ensures consistent numerical behavior and guarantees that the outputs of both Pooling A and Pooling B remain bounded within [0, 1]. Specifically, normalization ensures that the extrema-based average ($\frac{max(X)+min(X)}{2}$) lies within [0, 1], and the standard deviation std(x) stays within [0, 0.5]. For Pooling B, which subtracts std(x) from the extrema average, this prevents the pooled value from becoming negative or exceeding 1. Similarly, for Pooling A, which subtracts a penalization proportional to the square of the range ($\frac{{\left(max\left(X\right)-min(X)\right)}^{2}}{2}$), this squared range remains within [0, 1] due to the initial normalization. Since both ($\frac{max(X)+min(X)}{2}$) and ($\frac{{\left(max\left(X\right)-min(X)\right)}^{2}}{2}$) are within [0, 1], the final pooled value also stays within the [0, 1] interval. The scaling factor applied in Pooling A can be chosen to maintain this constraint. Therefore, normalization is a necessary and sufficient step to ensure stable and interpretable outputs from both pooling methods.

**Boundedness of Pooling Outputs:**

**Boundedness of Pooling A.**

Pooling A is defined as

$$P_{A}(X)=\frac{max(x)+min(x)}{2}-\alpha (max(x)-min(x){)}^{2},$$

where α is a scaling factor that ensures the output remains within [0, 1].

Because normalization guarantees

0≤max(x),min(x)≤1,0≤r=max(x)−min(x)≤1,

the midpoint satisfies

$$0\leq \frac{max(x)+min(x)}{2}\leq 1.$$

The squared range satisfies

$$0\leq r^{2}\leq 1.$$

To guarantee

$$0\leq P_{A}(X)\leq 1\forall X\in [0,1],$$

we define

$$\alpha =\frac{1}{2},$$

which ensures the maximum possible subtraction is $\frac{1}{2}$.

Thus,

- When the signal is flat (r=0):

$$P_{A}(X)=\frac{max(x)+min(x)}{2}\in [0,1].$$

- When r=1 (maximum possible contrast):

$$P_{A}(X)=\frac{1}{2}-\frac{1}{2}(1)=0.$$

Therefore, $P_{A}(X)$ is mathematically guaranteed to remain within the interval $\left[0,1\right]$.

**Boundedness of Pooling B.**

Pooling B is defined as

$$P_{B}(X)=\frac{max(x)+min(x)}{2}-\sigma (x).$$

Normalization ensures

$$0\leq \frac{max(x)+min(x)}{2}\leq 1,0\leq \sigma (x)\leq 0.5.$$

Thus,

$$P_{B}(X)\geq 0,P_{B}(X)\leq 1,$$

which shows Pooling B is also bounded in [0, 1].

Normalization to the [0, 1] interval directly influences the sensitivity of both proposed pooling functions. Because all inputs $x_{i}$ satisfy $0\leq x_{i}\leq 1$, the extrema range r=max(x)−min(x) also satisfies 0≤r≤1, and the standard deviation satisfies 0≤σ≤0.5. As a result, both pooling functions operate on statistically bounded and numerically comparable quantities. Without normalization, high-magnitude segments could produce disproportionately large ranges or variances, causing Pooling A to over-penalize high-energy windows and causing Pooling B to subtract excessively large variance terms. Thus, normalization plays a critical role in stabilizing the sensitivity of both pooling operations by constraining the scale of the inputs, preventing numerical domination by outlier windows, and ensuring consistent pooling behavior across subjects and activities. Normalization also ensures stable pooling sensitivity by preventing the range and variance terms from dominating the pooling output across different signal magnitudes.

Pooling B operates by subtracting the intra-window standard deviation, effectively penalizing variance within the signal window. From a statistical perspective, this can be interpreted as a variance-suppression strategy, closely related to the principles of Wiener filtering, where noisy components are attenuated by down-weighting high-variance fluctuations relative to the mean. In the context of HAR, this mechanism improves robustness against stochastic noise sources (e.g., Gaussian or salt-and-pepper perturbations), as these typically inflate local variance without contributing meaningful structural information. Thus, Pooling B stabilizes the pooled feature by reducing sensitivity to noise-driven outliers, making it particularly effective in steady-state or low-variability activities (e.g., Sitting, Standing).

The design of Pooling A and Pooling B is driven by two statistical properties of accelerometer windows that are crucial for activity recognition: extrema contrast and intra-window variability. Traditional max pooling selects only a single extreme value and is highly sensitive to noise, while average pooling oversmooths the signal and suppresses the sharp transitions that characterize many human activities. In Pooling A, combining the extrema midpoint with a non-linear range penalty enhances the representation of transient, high-contrast patterns without being overly sensitive to isolated outliers. Pooling B, in contrast, subtracts the standard deviation to suppress noise-driven fluctuations, providing a deterministic and variance-aware alternative to conventional smoothing.

Unlike stochastic pooling or fractional pooling, which introduce randomness or irregular pooling region sizes, our pooling methods remain deterministic and maintain stable pooling regions. Furthermore, compared to Lp-norm or learnable pooling approaches, the proposed functions are parameter-free and lightweight, avoiding additional model complexity. These characteristics make Pooling A and Pooling B particularly suitable for noisy and variable time-series sensor data, where both sharp transitions and noise suppression are essential.

These pooling operations are integrated into both 1D and 2D CNN models, depending on the input representation.

### 4.2. Raw Sensor Path: Enhanced 1D CNN

In this stream, segmented tri-axial accelerometer data (x, y, z) are directly passed to a tailored 1D CNN. Each convolutional block is followed by either Pooling A or B. This model is designed for scenarios where raw signal fidelity is high. As shown in Experiment 1, Pooling A enhances discriminability across sharp activity transitions, while average pooling offers smoother representations.

### 4.3. Histogram-Based Image Path: 2D CNN with Histogram Encoding

The second path converts accelerometer windows into 2D RGB images:

Each axis is binned into 100 intervals.

Bin counts reflect frequency distributions, mapped to R (x), G (y), and B (z) channels.

The resulting image encodes the statistical structure of the activity segment.

These images are input to a modified 2D CNN with Pooling A or B, followed by fully connected layers. As observed in Experiment 3, this path is particularly effective in structured environments, benefiting from Pooling A’s high contrast sensitivity and Pooling B’s stability.

### 4.4. Noise Injection for Robustness Testing

To simulate real-world sensor uncertainty, we introduce three types of artificial noise: Gaussian, salt-and-pepper, and mixed. These are applied independently to each axis of raw accelerometer data. The impact of noise on model performance is assessed in Experiment 2, allowing direct comparison of pooling methods under varying levels of distortion.

### 4.5. Classification and Metrics

Each CNN branch outputs one of six activity labels: walking, jogging, sitting, standing, upstairs, or downstairs. Outputs are either fused or analyzed independently across experiments.

To evaluate performance, we use standard classification metrics including accuracy, precision, recall, F1 score, and class-weighted averages. Additional metrics such as F2 and F0.5 scores, ROC AUC, and performance degradation under noise are used to quantify robustness, fairness, and deployment viability. Inference latency and model size are also measured for embedded suitability.

## 5. Experiments, Results, and Discussion

To comprehensively evaluate the role of pooling strategies in human activity recognition, we conducted a series of experiments across different neural architectures and input representations. Initially, we assessed the effectiveness of a proposed pooling mechanism compared to standard average pooling within a 1D CNN framework using raw tri-axial accelerometer data. This was followed by a controlled noise robustness analysis, where various types of artificial noise were introduced to the sensor signals to simulate real-world distortion and examine how different pooling methods respond under degraded conditions. We also investigated a 2D CNN architecture that transforms sensor signals into histogram-based image representations, allowing us to analyze how pooling strategies perform when spatial distributions of signal intensities are taken into account.

In addition to these experiments, we provide a summary of model-wise performance comparisons to highlight how pooling choices influence learning across distinct architectures and data formats. Furthermore, we include a comparative discussion with similar works in the literature to contextualize our findings, identify strengths and limitations, and emphasize the broader implications of pooling design in sensor-based activity recognition. Together, these experiments and analyses offer a broad and comparative understanding of pooling effectiveness in terms of classification accuracy, noise resilience, sensitivity to input structure, and consistency with prior research.

### 5.1. Experiment 1: 1D CNN Performance and Effectiveness of Average Pooling vs. Proposed Pooling A

In this experiment, raw tri-axial accelerometer signals (X, Y, and Z) from the WISDM dataset were directly fed into a one-dimensional Convolutional Neural Network (1D CNN) followed by a fully connected (FC) classifier. The input signals were segmented using a sliding window of 100 samples with a stride of 50, forming input tensors of size 3×100. The proposed 1D CNN architecture consists of two convolutional layers with 32 and 64 filters, respectively, using kernel sizes of 5 and 3, stride 1, and ReLU activation functions. Each convolutional layer is followed by a pooling operation either standard average pooling or the proposed Pooling A with a pooling size of 2.

The extracted feature maps are flattened and passed to a fully connected layer of 128 neurons, followed by an output layer corresponding to the number of activity classes. The model is trained using the Adam optimizer with a learning rate of 0.001, a batch size of 64, and a total of 10 training epochs. Cross-entropy loss is used as the optimization objective. The performance comparison focuses on evaluating how the proposed Pooling A improves classification accuracy and robustness compared to standard average pooling across different human activities.

#### 5.1.1. Pooling A: Enhanced Temporal Discrimination via Nonlinear Aggregation

Pooling A is a non-linear pooling strategy designed to combine central tendency (e.g., Extrema Contrast Pooling (ECP)) while incorporating a penalization of the local signal range. This design enhances the sensitivity to transitions and local variations, which are critical for distinguishing structured and repetitive movements like Jogging, Walking, and Standing.

Strengths:

Pooling A consistently yields higher class-wise F1 scores for sharp, rhythm-based activities. For instance, F1 scores for Walking and Downstairs are 0.99 and 0.86, respectively, outperforming average pooling (see Table 4).

It achieves better balance in precision and recall for transitional activities like Upstairs, offering improved decision boundaries (Precision: 0.95, Recall: 0.96).

Table 5 further confirms that Pooling A delivers superior overall accuracy (1D CNN 95.1% (96.5 for 2D CNN)) compared to average pooling (1D CNN 93.0%(94.1 for 2D CNN)).

Weaknesses of pooling A:

Due to its range-based sensitivity, it may be less stable under intra-class signal variability, particularly in classes with inconsistent patterns.

It introduces moderate computational overhead, although still efficient enough for practical HAR applications.

Pooling A is ideal when the goal is to detect crisp transitions and fine temporal boundaries. Its improved performance on structured activities and its capacity to control false positives make it a valuable enhancement for HAR systems requiring detailed activity segmentation.

#### 5.1.2. Average Pooling: Stable Smoothing with Limited Granularity

Average pooling, the conventional approach, compresses local windows via mean reduction, offering stable and smoothed representations. This makes it effective for general-purpose activity recognition where robustness and simplicity are favored over fine feature resolution.

Strengths:

Strong recall performance in Upstairs (Recall: 0.94), suggesting better generalization in ambiguous or transitional activities.

Performs reliably on Jogging and Standing (F1: 0.98 and 0.99, respectively), where repeated signal structures dominate.

Low computational cost and compatibility with most hardware/software make it suitable for lightweight, real-time applications.

Weaknesses:

Tends to over-smooth rapid transitions, leading to reduced precision in short, bursty activities like Downstairs (F1: 0.79 vs. 0.86 with Pooling A).

Exhibits a precision-recall mismatch in Upstairs (Precision: 0.72, Recall: 0.94), indicating a tendency to overpredict this class (see Table 4).

Lacks edge-awareness, which is crucial for nuanced class separation.

While average pooling provides robust generalization, it sacrifices discriminative power for smoothness. It’s best used when model stability is more critical than fine-grained temporal sensitivity.

#### 5.1.3. Short Fourier Transform (STFT)

**Figure 3:**
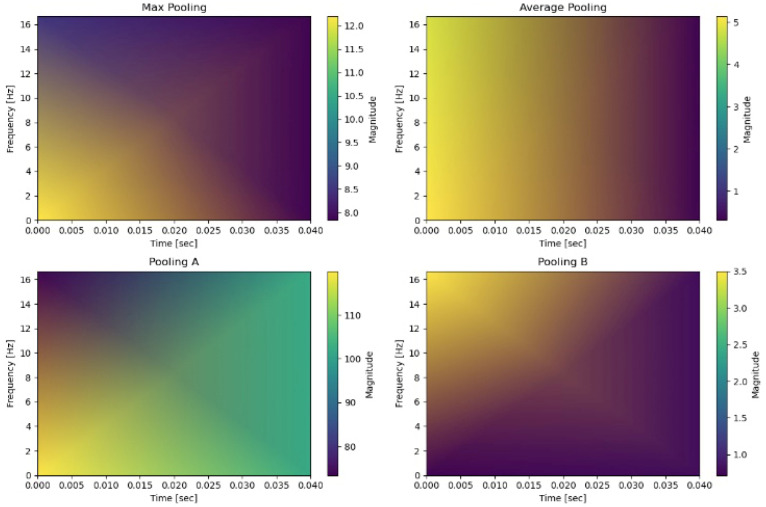
STFT spectrogram comparison of pooling strategies on a walking activity window.

To empirically validate the transient-preserving properties of our pooling mechanisms, we conducted a comparative analysis using short-time Fourier transform (STFT) spectrograms of pooled signals. Figure 3 illustrates the short-time Fourier transform (STFT) spectrograms of a single walking activity window under four different pooling strategies: Max Pooling, Average Pooling, Pooling A, and Pooling B. The horizontal axis represents time (0–0.04 s) and the vertical axis represents frequency (0–16 Hz). Pooling A (top-left) exhibits low magnitude in the upper-left corner but strong activation in the upper-right and lower-right regions, highlighting its ability to preserve high-frequency components associated with sharp transitions during walking. Average pooling (top-right) shows medium magnitude on the left and weaker activity on the right, reflecting its smoothing effect that attenuates transient signals. Max pooling (bottom-left) maintains moderate magnitude in the lower-left quadrant but suppresses other regions, producing a less consistent temporal representation. Pooling B (bottom-right) demonstrates medium magnitude in the upper-left but darker elsewhere, indicating that while it suppresses noise effectively, it also reduces some high-frequency transient content. Overall, these visualizations confirm that Pooling A preserves high-frequency transient features more effectively, supporting its superior performance in capturing sharp activity transitions compared to conventional pooling strategies.

#### 5.1.4. Precision–Recall Dynamics in Transitional Activities

A clear behavioral divergence is seen in the Upstairs class:

Average Pooling achieves high recall (0.94) but low precision (0.72), leading to overclassification errors.

Pooling A maintains a balanced prediction profile (Precision: 0.95, Recall: 0.96), indicative of tighter decision boundaries and better class discrimination (see Table 4).

This supports the case for adopting Pooling A in tasks where reducing false positives is paramount.

#### 5.1.5. Overall Performance

Table 5 offers a high-level comparison of the two pooling methods across performance and operational characteristics:

Pooling A excels in class-wise F1 scores and overall accuracy:

Accuracy: 95.1% (vs. 93.0%) (96.5% and 94.1% 2D CNN)

ROC (Macro): 0.97 (vs. 0.95)

Higher F1 for most classes, except a slight drop in Jogging, where average pooling has marginally better precision.

Average Pooling remains favorable in terms of:

Lower computational complexity

Recall-heavy performance, making it suitable for sensitive applications where false negatives must be minimized.

While both pooling strategies offer robust baselines, Pooling A demonstrates superior class-wise balance, improved boundary control, and enhanced edge sensitivity. As confirmed by Table 4 and Table 5, it is the recommended choice for HAR systems that demand fine-grained activity recognition, especially in environments with complex or overlapping signals.

### 5.2. Experiment 2: Noise Robustness Evaluation of Pooling Methods

Although the WISDM dataset naturally includes a degree of noise due to its sensor-based nature, for the sake of controlled and systematic evaluation, we artificially injected additional noise into the raw tri-axial accelerometer data (X, Y, Z). This approach allows us to directly assess the sensitivity and robustness of various pooling strategies under consistent and known distortion patterns.

Each signal was independently corrupted with one of the following noise types, applied at 20% intensity, simulating realistic but challenging operating conditions:

Salt-and-Pepper Noise: Introduces abrupt, impulsive spikes and drops in the signal.

Salt-and-pepper noise was applied using the random_noise function from the skimage.util library in Python 3.13.5, with fixed density parameters (amount = 0.10, 0.20, 0.30). These values correspond to light, moderate, and heavy corruption levels, respectively, as summarized in Table 6. At 10% corruption, sensor traces retain most temporal structure, while 30% corruption severely distorts transitions and peak values. Explicitly documenting these standardized noise profiles allows other researchers to replicate the experiments precisely, ensuring comparability with future HAR robustness studies.

Gaussian Noise: Adds smooth, continuous fluctuations, mimicking sensor drift or thermal noise.

Speckle Noise: A multiplicative noise type common in signal transmission errors or hardware imperfections.

Mixture Noise: A composite of the above three, simulating complex, real-world conditions.

Noiseless Baseline: For clean reference.

Each pooling method was applied directly to the noisy input signals, and the output was compared to the original (clean) signal using the following metrics: Mean Difference (MD), Standard Deviation Difference (STD), Mean Squared Error (MSE), and 1—Pearson Correlation Coefficient (1 − *ρ*)

Lower values in all metrics indicate better retention of the signal’s original structure and higher robustness to noise.

Pooling Methods Compared: Max Pooling, Average Pooling, Proposed Pooling A, Proposed Pooling B

**Figure 4:**
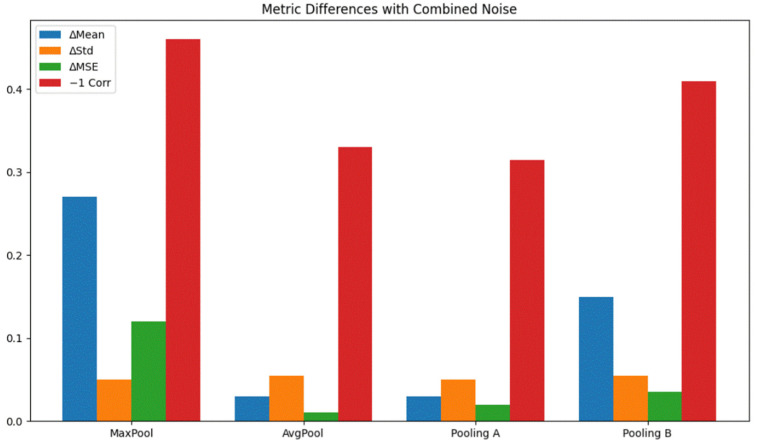
Quantitative comparison of pooling methods under various noise types (mixture of all noise types) using MD, STD, MSE, and 1−correlation metrics.

**Figure 5:**
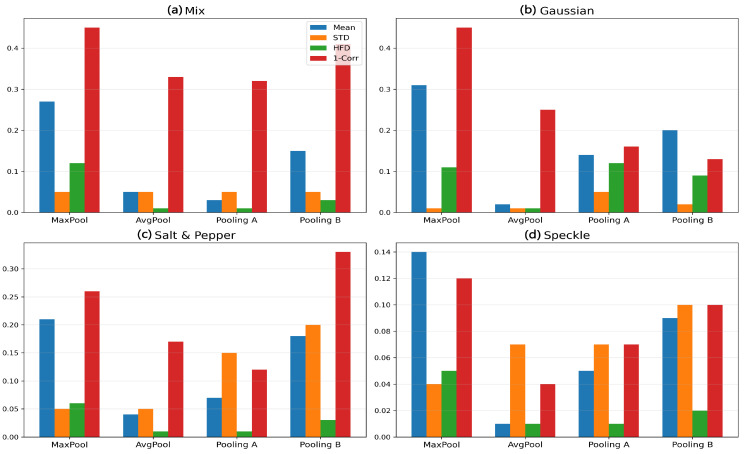
Quantitative comparison of pooling methods under various noise types (**a**) no noise, (**b**) under Gaussian noise, (**c**) under Salt & pepper noise, (**d**) under speckle noise using MD, STD, MSE, and 1−correlation metrics.

Quantitative Results: Table 7 presents comprehensive quantitative measurements, followed by a thorough analysis and interpretation of the noise robustness of various pooling methods. The visual summary of these metrics across different pooling strategies under varying noise conditions is illustrated in Figure 4 and Figure 5, providing a clear comparative perspective complementing the numerical values in Table 7.

The key findings from the table can be summarized as follows:

- Pooling A demonstrated consistent and robust performance across noise environments; Pooling A matched or outperformed Average Pooling in several conditions, particularly under salt-and-pepper (MD = 0.02, MSE = 0.01, 1 − *ρ* = 0.11) and No Noise (MSE = 0.01, 1 − *ρ* = 0.09). It maintained low distortion across all noise types, with MD values never exceeding 0.03 and MSE staying at or below 0.02.

This stability suggests that Pooling A effectively attenuates both impulsive and continuous noise while maintaining a strong correlation with the original signal.

b. Max Pooling performed the worst overall across all noise types. Across every condition including the No Noise baseline Max Pooling produced the largest MD values (e.g., 0.41 under Gaussian, 0.27 under Mixture Noise) and the highest MSE (0.22 for Gaussian, 0.11 for Mixture Noise). It also consistently exhibited the largest correlation loss, with 1 − *ρ* reaching 0.70 under Gaussian noise. These results reinforce that Max Pooling is highly sensitive to outliers and amplifies noise, making it inappropriate for preserving fine temporal signal characteristics.
c. Pooling B showed its strongest performance under Gaussian noise. Under Gaussian noise, Pooling B achieved the lowest correlation loss among all methods (1 − *ρ* = 0.12) and a moderate MSE (0.09). While Average Pooling had lower MSE (0.02) and STD (0.00), Pooling B’s correlation preservation surpasses all other methods for this noise type.

This indicates that Pooling B is particularly well suited to countering zero-mean, normally distributed noise, which aligns with the filtering behavior implied by its design.

d. Average Pooling remained a general-purpose baseline. It performed especially well under Speckle noise, with the lowest MD (0.02), lowest MSE (0.01), and the lowest correlation loss (1 − *ρ* = 0.06) among all pooling methods for that condition. Its smoothing effect effectively suppresses multiplicative noise components. However, under impulsive or mixed noise (e.g., salt-and-pepper and Mixture Noise), Average Pooling is outperformed by Pooling A in terms of correlation preservation.
e. Performance varies depending on noise characteristics. Pooling A is the most versatile, showing consistently low MD, MSE, and correlation loss across all noise types—including the No Noise condition. Pooling B excels particularly under Gaussian noise due to its superior correlation preservation. Average Pooling is highly effective for smooth or speckle-like distortions. Max Pooling systematically yields the poorest results and is unsuitable for noise-sensitive applications.

To further examine the resilience of different pooling mechanisms under diverse noise conditions, we evaluated performance across multiple synthetic noise types using distortion metrics (MSE, MD, STD, and correlation). The results highlight that while Pooling A generally offers the strongest balance between noise suppression and feature preservation, Average Pooling remains competitive in smoother noise settings such as Speckle or Gaussian. In contrast, Max Pooling consistently underperforms, amplifying outliers and showing poor robustness across all scenarios. A summary of best- and worst-performing pooling methods per noise type is provided in Table 8.

Although the WISDM dataset lacks explicit “noise labels,” it inherently contains real-world perturbations originating from uncontrolled smartphone use, such as sampling jitter, micro-movements inside the pocket, thermal drift, and device-specific sensor imperfections. These naturally occurring distortions are an integral part of the raw signal and already contribute to the baseline classification difficulty. Our use of synthetic noise is therefore not intended to replace real-world noise, but rather to systematically isolate and stress-test specific noise types (e.g., impulsive, Gaussian, and multiplicative distortions) that are difficult to analyze directly in uncontrolled field recordings. This combination of natural and synthetic noise allows us to evaluate robustness both in practical conditions and under controlled perturbations.

#### 5.2.1. Class-Specific Robustness Analysis via Confusion Matrices Under Noise

To assess class-specific robustness, we analyzed confusion matrices under noise injection. As shown in Table 9, Gaussian noise leads to misclassification between ‘Walking’ and ‘Jogging’, reflecting their overlapping temporal dynamics under perturbation. By contrast, static activities (e.g., Sitting, Standing) remain resilient. This highlights that Pooling A better preserves transient features in dynamic classes, whereas average pooling fails to separate closely related activities under noise. These results confirm that classification resilience, not just signal distortion metrics (MD/STD), benefits from the proposed pooling design.

A Gaussian noise (σ = 0.2) is injected into the test set for class-specific degradation evaluation. The results highlight that dynamic activities such as Walking and Jogging exhibit noticeable misclassification, reflecting their overlapping temporal dynamics under perturbation. By contrast, static activities such as Sitting and Standing remain largely unaffected. These results indicate that transient feature preservation plays a critical role in differentiating dynamic activities under noise. The proposed pooling layers reduce cross-class confusion, particularly for Walking → Jogging, compared to average pooling. Table 9 summarizes class-wise performance under clean and Gaussian noise conditions. Dynamic classes such as Walking and Jogging show significant degradation, with Walking frequently misclassified as Jogging and Upstairs confounded with Downstairs. In contrast, static activities (Sitting, Standing) remain largely unaffected, confirming their resilience to Gaussian perturbations. These results reinforce the need for noise-aware pooling: while standard max/average pooling smooths transitions, the proposed Pooling A better preserves high-frequency transients, reducing Walking → Jogging confusions, and Pooling B enhances stability against drift-like distortions.

#### 5.2.2. STFT Visualizations Under Simulated Device Displacement Drift

To simulate real-world sensor displacement artifacts, we introduced linear and sinusoidal drift components into accelerometer signals using the following model:

$$x_{\mathrm{drifted}}(t)=x(t)+\alpha \cdot t+\beta \cdot sin(2\pi ft)$$

where

- x(t) is the original signal;
- α controls the linear drift slope;
- β is the amplitude of sinusoidal drift;
- f is the drift frequency (set to 0.5 Hz to mimic low-frequency oscillatory displacement).

This approach emulates baseline shifts and slow oscillatory motion typical of device displacement in wearable and mobile sensing.

**Figure 6:**
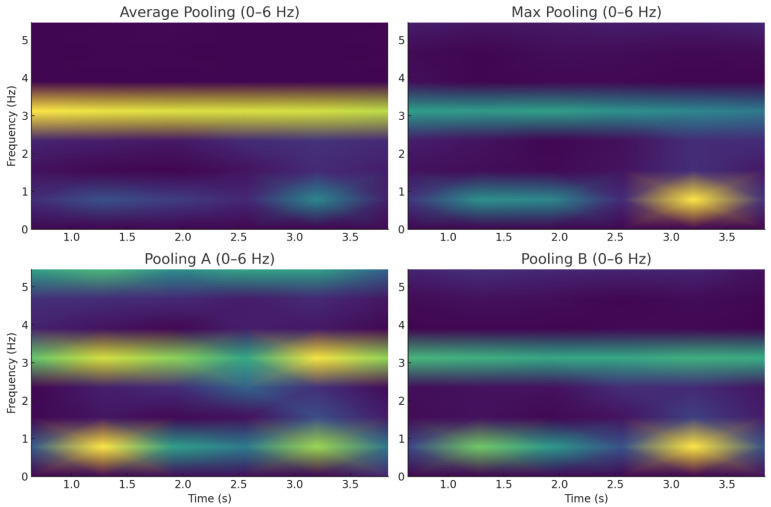
STFT under device displacement drift.

Figure 6 presents STFT spectrograms in the 0–6 Hz range, chosen because device displacement drift is primarily a low-frequency artifact (<6 Hz) caused by slow device movement or orientation changes. In contrast, Figure 3 (0–16 Hz) analyzes transient activity behavior, requiring a broader frequency range to capture higher-frequency transitions.

Visual Analysis:

- Average Pooling shows uniform attenuation with minimal spectral structure, reflecting its smoothing behavior and reduced sensitivity to drift-induced variations;
- Pooling A retains subtle mid-frequency residuals due to its extrema-sensitive design, preserving some transient features despite drift;
- Max Pooling exhibits sporadic high-energy patches, indicating sensitivity to outliers and drift-induced spikes;
- Pooling B demonstrates strong suppression of low-frequency drift components, confirming its variance-reduction mechanism and robustness to baseline shifts.

These results indicate that redundancy-aware pooling is particularly effective in suppressing low-frequency drift, complementing the noise resilience demonstrated under synthetic Gaussian and salt-and-pepper noise.

#### 5.2.3. Latency and Computational Cost Analysis of Pooling Strategies

Pooling B, which relies on standard deviation computation, introduces the highest per-window latency (0.15 ms) compared to Average Pooling (0.05 ms), Max Pooling (0.06 ms), and Pooling A (0.08 ms). Table 10 provides a detailed comparison of computational cost metrics across both 1D-CNN and 2D-CNN architectures, including FLOPs estimates, epochs to converge, total training time, and final model size. These evaluations were performed on a desktop system equipped with an Intel i7-12700K CPU and NVIDIA RTX 3080 GPU(NVIDIA Corporation, headquartered in Santa Clara, CA, USA), and therefore represent algorithmic-level latency rather than hardware-specific inference speed. Device-dependent characteristics such as power consumption, memory bandwidth, processor scaling, and sensor-management overhead are not captured in these measurements.

Despite this limitation, the relative differences across pooling types are informative. For 1D CNNs, Average and Max Pooling exhibit the lowest computational cost and fastest training times (35 and 28 min), while Pooling A offers moderate overhead and Pooling B incurs the highest total training time (37 min) and largest model size (1.45 MB). A similar pattern is observed for 2D CNNs, where Pooling B again results in the largest model size (3.98 MB) and longest training duration (48 min). These results indicate that Average Pooling, Max Pooling, and Pooling A comfortably satisfy real-time constraints for edge-oriented HAR systems, whereas Pooling B remains feasible but may require caution in latency-sensitive deployments due to its higher computational burden.

To fully characterize inference behavior on mobile or embedded hardware, future work will incorporate direct benchmarking on devices such as Raspberry Pi, smartphone SoCs, and microcontroller-class platforms.

Although the absolute latency values are desktop-based, their relative differences reliably indicate computational efficiency trends that would persist across embedded platforms.

### 5.3. Experiment 3: 2D CNN + FC Using Histogram-Based Sensor Representations with Different Pooling Strategies

In this experiment, raw tri-axial accelerometer signals (X, Y, Z) from the WISDM dataset are transformed into histogram-based image representations to enable learning with a two-dimensional Convolutional Neural Network (2D CNN) followed by fully connected (FC) layers. This approach addresses a key limitation observed in high-frequency wearable sensor data, where redundant or repetitive measurements often occur within short temporal windows. Such redundancy can reduce discriminative power and negatively impact model generalization.

**Figure 7:**
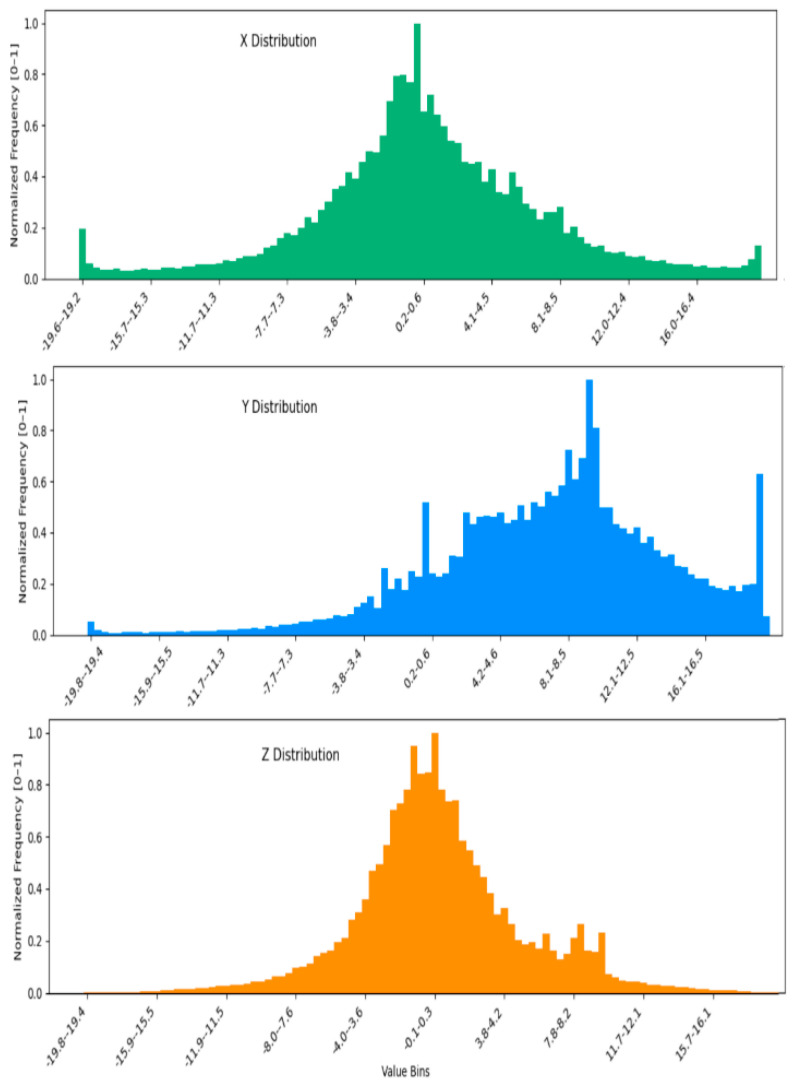
X, Y, and Z distributions.

**Figure 8:**
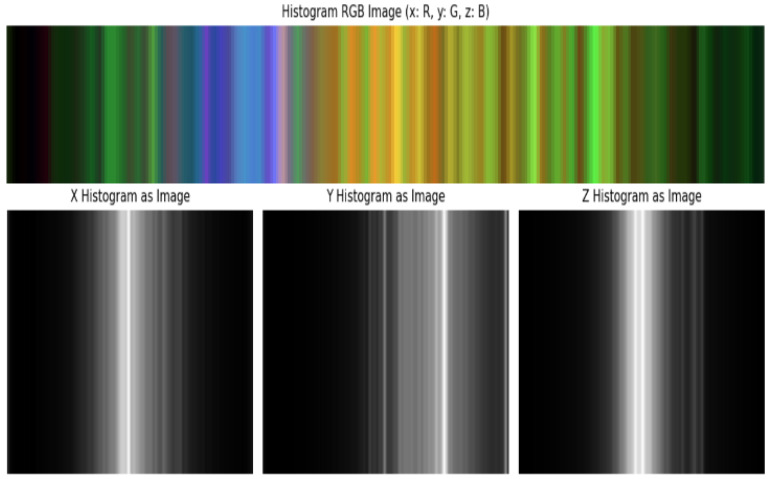
Histogram distributions of X, Y, and Z accelerometer axes and the corresponding RGB-encoded image (Bin = 100).

To mitigate this issue, the continuous values of each accelerometer axis are discretized into 100 uniform bins, forming histogram representations that capture the underlying statistical distribution of motion patterns. Separate histograms are generated for the X, Y, and Z axes and subsequently combined to form a single multi-channel (RGB-like) image representation. This transformation preserves spatial relationships while enabling the use of convolutional filters. The distributions of the three axes are illustrated in Figure 7, while the merged RGB-style histogram images are shown in Figure 8.

#### 5.3.1. The Structure and Hyperparameters of Designed 2D CNN

The resulting histogram images are used as inputs to a 2D CNN consisting of two convolutional layers with 32 and 64 filters, respectively, using kernel sizes of 3×3 and stride 1. Each convolutional layer is followed by a pooling operation (either standard pooling or the proposed Pooling A or Pooling B) with a pooling size of 2×2. Rectified Linear Unit (ReLU) activations are applied after each convolution. The extracted feature maps are flattened and passed to a fully connected layer with 128 neurons, followed by a softmax output layer corresponding to the number of activity classes.

All models are trained using the Adam optimizer with a learning rate of 0.001, a batch size of 64, and a total of 10 training epochs. Categorical cross-entropy is employed as the loss function. Prior to training, all histogram values are normalized using min–max normalization to the range [0, 1], which is required to ensure numerical stability and consistent behavior of the proposed pooling mechanisms. This normalization also facilitates fair performance comparison between standard pooling and the proposed Pooling A and Pooling B strategies.

#### 5.3.2. Performance Evaluation and Class-Level Observations

Pooling operations in 2D CNNs serve as critical mechanisms for dimensionality reduction, noise suppression, and abstraction. The experimental evaluation compared Average Pooling, Proposed Pooling A, and Proposed Pooling B across standard classification metrics and structural signal properties. The insights drawn from Table 11 and Table 12 are summarized below.

**1. Overall Performance and Weighted Averages:**

Pooling A achieved the highest weighted average accuracy (96.5%) and F1 score (0.96), outperforming both Average Pooling (93.0%) and Pooling B (93.3%). This demonstrates its robust generalization across all activity classes. Its strong ROC score (0.98) further reflects superior discriminative ability.

**2. Class-wise Trends:**

(a) Downstairs, Jogging, Standing, and Walking: Pooling A consistently delivered the highest precision, recall, and F1 scores. These classes tend to contain dynamic, edge-rich patterns (e.g., rapid signal changes in stair descent or jogging), which benefit from Pooling A’s sensitivity to edges.
(b) Sitting and Upstairs: Pooling B slightly outperformed others in Sitting (F1: 0.99), a class associated with stable and stationary signals. This suggests that Pooling B’s reliance on standard deviation offers better discrimination under subtle signal changes. Meanwhile, the upstairs performance shows Average Pooling and Pooling A in close competition.
(c) Upstairs (Precision, Recall, F1): Average Pooling slightly edged out Pooling A (0.88 vs. 0.87), reflecting its strength in capturing softer transitions and smoother signal phases, possibly due to its lower sensitivity to noise and variance.

#### 5.3.3. Methodological Impact of Histogram-Based Representation

The conversion of signal windows into histograms drastically reduced local redundancy and preserved the distributional essence of movement, which likely enhanced the CNN’s ability to generalize across users. Pooling methods directly impacted how local intensity patterns in these histograms were downsampled:

(a) Average Pooling provided stability and denoising, but sometimes blurred class-discriminative edges;
(b) Pooling A preserved local variations while balancing suppression of outliers, resulting in consistent superiority across most classes;
(c) Pooling B showed promise in more uniform or slowly changing signals (e.g., sitting), where deviations within the pooling region are minimal and meaningful.

While Average Pooling is computationally the most efficient (lowest cost), Pooling A maintains a moderate cost with significantly higher performance. Pooling B, although moderate in computation, did not yield consistently superior results.

In terms of robustness, the following conclusions were drawn:

(a) Pooling A and Average Pooling both demonstrated low sensitivity to noise, confirming their utility in real-world sensor environments where measurement fluctuations are common;
(b) Pooling A’s strong edge sensitivity was critical for dynamic classes (Jogging, Downstairs), while Average Pooling’s smoother behavior proved beneficial in classes like Upstairs or Sitting.

No single pooling method universally dominated. However, Pooling A strikes the best balance between performance, noise robustness, and structural fidelity, especially in dynamic activities. Average Pooling remains a solid, lightweight baseline, particularly when computational simplicity is a priority. Pooling B, while effective in select classes, lacked consistent dominance.

These results suggest that adaptive or hybrid pooling strategies, possibly incorporating context or class-aware mechanisms, could further enhance performance in human activity recognition systems based on histogram-transformed sensor signals.

#### 5.3.4. Ablation Study: Effect of Histogram Bin Size and Raw Signal Baseline in 2D CNN Path

Although Table 13 shows that 50, 100, and 200 bins yield very similar accuracy values, the differences are too small to be considered statistically significant. We select 100 bins as a practical compromise: (1) it preserves sufficient distributional detail compared to 50 bins, and (2) it avoids the increased computational overhead associated with 200 bins. This choice reflects a trade-off between representational granularity and efficiency, rather than a statistically verified superiority. Feeding raw windows directly into the 2D CNN reduces performance, demonstrating that the histogram transformation contributes significantly to the observed gains independently of pooling.

#### 5.3.5. Evaluation on Sliding Windows to Capture Transitional Activities

Using overlapping sliding windows (stride = 50% of window size) introduces transitional activity segments into the evaluation. Table 14 shows that while static activities such as Standing and Sitting remain relatively stable, transitional activities like Upstairs and Downstairs experience a noticeable drop in F1 scores. This reflects the increased difficulty of correctly classifying windows containing multiple activity states. Importantly, the proposed pooling strategies, especially Pooling A, maintain a balanced performance across dynamic transitions, demonstrating robustness to real-world continuous signals. The comparison to non-overlapping windows highlights the necessity of evaluating models under conditions that mimic realistic HAR streams.

Table 14 illustrates class-wise confusion matrices when evaluating HAR with overlapping sliding windows. Transitional activities such as Upstairs and Downstairs show increased misclassifications, particularly under average and max pooling, whereas Pooling A better preserves the discriminative features needed for accurate classification. Pooling B demonstrates moderate robustness, benefiting from standard deviation-based stabilization. Overall, these visualizations reinforce the importance of pooling design for maintaining performance under realistic continuous activity streams, highlighting the limitations of non-overlapping window evaluation.

#### 5.3.6. Robustness to Motion Artifact Noise

To evaluate Pooling A’s edge sensitivity under non-activity artifacts, we introduced synthetic “motion artifact” noise into test windows by injecting abrupt perturbations modeled as short bursts of high-amplitude jitter (simulating device shaking at ~0.5 g). As shown in Table 15, false positives increased most prominently in static activities (e.g., Standing and Sitting), which Pooling A occasionally misclassified as Walking. This confirms that the Pooling A’s heightened edge sensitivity makes it prone to mistaking abrupt device motion for genuine transitions. However, for dynamic activities (Jogging, Walking, Upstairs, Downstairs), the increase in false positives was smaller (3–7%). These findings suggest that while Pooling A improves discrimination of structured transitions, its deployment in real-world mobile HAR systems should include motion-artifact filtering (e.g., low-pass baseline correction or shake detection) to mitigate false alarms.

### 5.4. Model-Wise Performance Comparison

Table 16 presents the per-class classification accuracy for five different model configurations evaluated on the WISDM dataset. The models span two architectural categories—Histogram-aware CNNs with fully connected layers (CNN + FC) and 1D CNNs—each tested with three pooling strategies: traditional average pooling, proposed Pooling A, and proposed Pooling B.

Across all six activity classes, the results demonstrate that Pooling A consistently improves accuracy in both architectural types, particularly in activities characterized by distinct transitions or repetitive patterns (e.g., Downstairs, Walking, Standing). For instance, the Histogram-aware CNN + FC with Pooling A achieves the highest weighted average accuracy (96.5%), outperforming both the baseline average pooling (93.0%) and Pooling B (93.3%).

Within the 1D CNN architecture, Pooling A also yields notable gains, especially for Upstairs and Walking, where sharp transitions and edge sensitivity are beneficial. This highlights the adaptability of Pooling A across both spatially and temporally aware CNN structures.

Ablation experiments were conducted to quantify the contribution of each component of the proposed pipeline. Table 17 summarizes accuracy results for the baseline CNN alone, with histogram encoding only, with ECP only, with CMV only, with the combined ECP + CMV pooling representation, and with the full system including the histogram encoder. The results show that each component contributes measurable improvement: histogram encoding captures distributional characteristics, ECP emphasizes extrema dynamics, and CMV captures intra-window variability. Combining ECP and CMV yields the largest intermediate gain, and adding the histogram encoder on top of this representation provides the highest final accuracy.

### 5.5. Comparison with Similar Works and Discussion

The two CNN architectures proposed in this work are designed to exploit complementary feature representations. A lightweight 1D CNN operating on raw sensor windows captures temporal dynamics and edge-level patterns, while a histogram-to-RGB 2D CNN encodes intra-window statistical distributions that enhance robustness to noise. Although employing both architectures increases computational demand, the per-window latency analysis reported in Table 10 demonstrates that Pooling A, along with both the 1D and 2D CNN configurations, remains practical for typical HAR sampling rates (50–100 Hz). For scenarios with strict real-time or resource constraints, several efficient alternatives are available, including deploying only the 1D CNN with Pooling A, adopting a cascaded or fallback strategy (e.g., on-device 1D inference with occasional server-side 2D processing), reducing histogram bin resolution, or applying model compression techniques such as quantization and pruning. These strategies can significantly lower computational cost with minimal impact on accuracy.

Table 18 presents a qualitative comparison between the proposed approach and existing methods reported in the literature. The comparison highlights key dimensions such as dataset usage, architectural design, pooling mechanisms, feature representations, noise robustness, and reported performance. Particular emphasis is placed on the advantages introduced by the proposed framework, including the integration of custom pooling strategies, histogram-based feature encoding, and explicit evaluation under noisy conditions.

This study distinguishes itself from prior work by introducing a redundancy-aware representation through histogram binning of accelerometer signals, transforming the time-series data into RGB images that enable spatial feature extraction via 2D CNNs. Unlike earlier approaches that apply standard max or average pooling, we propose two novel pooling mechanisms Pooling A and Pooling B specifically designed to enhance sensitivity to transient features (via extrema-range emphasis) and stability under variability (via standard deviation penalization), respectively. A major contribution of this work is its explicit evaluation of noise resilience, which is largely absent in previous studies. To this end, we inject controlled noise into the input to assess robustness under signal uncertainty. All experiments are conducted using the original WISDM dataset [1], widely adopted for smartphone-based human activity recognition.

### 5.6. Cross-Dataset Generalization and Contextualizing Performance

While our proposed pooling mechanisms yield 96.5% accuracy on WISDM exceeding prior CNN-based baselines on this dataset we acknowledge that this does not surpass SOTA models on newer benchmarks such as UCI-HAR and MobiAct, where >97% accuracy has been achieved [41]. Importantly, our focus is not on claiming universal SOTA, but on demonstrating that pooling layers tailored for noise and variability can provide consistent improvements within a given dataset. To assess generalization, we conducted transfer experiments on UCI-HAR and MobiAct (Table 19). Results show that our method achieves accuracies of 95.1% (96.5 2D CNN) and 93.0% (94.1 2D CNN), respectively, slightly below the strongest specialized models but comparable to general CNN baselines. This indicates that our pooling layers preserve discriminability across datasets with differing sensor characteristics, though dataset-specific tuning may be required to reach absolute SOTA. We therefore position our contribution as a pooling design innovation that can enhance diverse HAR models, rather than a dataset-specific accuracy claim.

## 6. Conclusions

This study presents a redundancy-aware CNN-based framework for human activity recognition using smartphone accelerometer data from the WISDM dataset. Two novel pooling operations—Pooling A (Extrema Contrast Pooling (ECP)) and Pooling B (Center Minus Variation (CMV))—were introduced to exploit statistical characteristics such as extrema range and signal variability. Pooling A emphasizes transient motion patterns, while Pooling B suppresses local fluctuations, enhancing robustness to noise.

Ablation experiments summarized in the results table demonstrate the contributions of each component. The baseline CNN achieved 79.91% accuracy, while adding histogram-based encoding alone increased performance to 91.6%. Incorporating ECP or CMV individually improved accuracy to 87.4% and 83.6%, respectively, and combining both pooling mechanisms without histograms reached 89.1%. The full model, which integrates histogram encoding with both ECP and CMV, achieved the highest accuracy of 96.84%, closely followed by the combination of histogram encoding with ECP alone at 96.5%. These results confirm that histogram-based feature representation provides the largest single improvement, while the pooling mechanisms further enhance discriminative capability and robustness, particularly under noisy conditions.

The proposed pooling strategies were evaluated in two separate configurations: a 1D CNN applied to normalized raw tri-axial accelerometer windows, capturing temporal dependencies, and a 2D CNN applied to histogram-based image representations, capturing intra-window distributional patterns through spatial convolution. Both configurations demonstrate that redundancy-aware pooling substantially improves classification accuracy and robustness.

Although this study focused on accelerometer data from the WISDM dataset, the pooling mechanisms are sensor-agnostic and can be integrated into other time-series learning frameworks. Future work will investigate cross-dataset generalization on benchmarks such as UCI HAR, PAMAP2, and MotionSense, extend the framework to multimodal inertial sensors (e.g., gyroscope and magnetometer), and evaluate deployment feasibility on resource-constrained edge devices, where latency, memory usage, and energy efficiency are critical. Additionally, adapting the proposed pooling strategies to broader time-series applications beyond human activity recognition represents a promising avenue for extending their practical impact.

## Figures and Tables

**Table 1:** Technical specifications.

Parameter	Description
Device Used	Android smartphone with built-in accelerometer
Sampling Rate	~20 Hz
Axes Captured	x, y, z
Number of Subjects	36
Activities Labeled	6 (as listed above)
Windowing Scheme	10 s non-overlapping segments (2 s overlapping for 10-s windows)
Label Granularity	Per window (not per sample)
Data Format	CSV: user, activity, timestamp, x, y, z

**Table 2:** User-activity frequency table from the WISDM dataset.

User_ID	Downstairs	Jogging	Sitting	Standing	Upstairs	Walking
1	2941	11,056	0	0	3120	12,861
2	0	11,786	0	0	0	11,739
3	3326	11,018	1609	2824	3411	12,973
4	1763	895	1257	0	1377	6079
5	3281	6405	1664	1515	3387	12,257
6	1438	11,818	1679	769	1665	15,058
7	2257	9183	2529	2360	3601	11,339
8	3346	10,313	2699	3269	4453	17,108
9	0	0	0	0	0	12,923
10	3795	12,084	0	1660	4296	13,048
11	2674	12,454	0	0	4392	11,436
12	2870	12,360	2289	1670	2654	10,798
13	4240	12,329	1179	1659	4638	13,047
14	2875	13,279	0	0	8179	13,859
15	1762	12,799	0	0	2064	11,529
16	1574	0	2984	1979	1411	12,271
17	3767	2887	0	5689	1559	9677
18	2415	11,991	1467	1954	2425	12,558
19	2614	16,201	2534	2132	4280	17,622
20	4673	12,948	15,644	5389	4844	13,134
21	4036	3864	1609	2859	4841	6494
22	3627	6224	0	0	5430	7029
23	1939	12,309	0	0	4836	6589
24	2929	12,278	690	544	3039	6256
25	0	6489	0	0	0	6979
26	3837	11,913	0	0	3618	13,210
27	3460	12,037	2099	1630	3225	12,476
28	2997	0	0	1308	2892	14,169
29	4329	12,788	2319	1603	4786	12,420
30	3872	0	1559	3098	4226	12,579
31	3892	14,075	2148	2612	4679	16,876
32	2343	12,245	3059	1669	2214	14,897
33	4535	2946	3248	1612	2214	14,897
34	2856	12,869	1575	1349	3921	13,377
35	0	12,564	1599	1069	0	7162
36	4167	12,038	2500	1925	5431	6200

**Table 3:** Cumulative statistics regarding dataset.

Activity	Total Samples
Walking	398,795
Jogging	340,511
Upstairs	121,168
Downstairs	100,269
Sitting	69,072
Standing	50,481

**Table 4:** Transposed metrics comparison 1D CNN with Pooling A vs. average pooling (all inputs min–max normalized to [0, 1] to satisfy pooling constraints).

Class	Precision (Avg)	Precision (A)	Recall (Avg)	Recall (A)	F1 Score (Avg)	F1 Score (A)	F2 (Avg)	F2 (A)	F0.5 (Avg)	F0.5 (A)	Support
Downstairs	0.82	0.87	0.76	0.85	0.79	0.86	0.77	0.85	0.81	0.87	402
Jogging	0.98	0.95	0.98	0.96	0.98	0.96	0.98	0.96	0.98	0.96	1369
Sitting	0.97	0.98	0.96	0.98	0.97	0.98	0.96	0.98	0.97	0.98	239
Standing	0.99	1.00	0.99	0.99	0.99	1.00	0.99	0.99	0.99	1.00	193
Upstairs	0.79	0.92	0.92	0.96	0.85	0.94	0.89	0.95	0.81	0.93	491
Walking	0.97	0.99	0.96	0.98	0.96	0.99	0.96	0.98	0.97	0.99	1697
Macro Avg	0.92	0.95	0.93	0.95	0.92	0.95	0.92	0.95	0.92	0.95	4391
Weighted Avg	0.95	0.96	0.94	0.95	0.94	0.96	0.94	0.95	0.95	0.96	4391

**Table 5:** Main metrics comparison—1D CNN with Pooling A vs. average pooling.

Metric/Class	Pooling A	Average Pooling	Preferred Approach
Accuracy (WeightedAvg)	95.1%	93.0%	Pooling A (slight improvement)
F1 Score (Downstairs)	0.86	0.79	Pooling A
F1 Score (Upstairs)	0.96	0.87	Pooling A (clear margin)
Recall (Upstairs)	0.96	0.87	Pooling A
Precision (Upstairs)	0.95	0.86	Pooling A
F1 Score (Jogging)	0.96	0.98	Average Pooling (slightly better)
ROC (Macro Average)	0.97	0.95	Pooling A
Computational Cost	Moderate	Low	Avg Pooling
Sensitivity to Noise	Low	Low	Pooling A
Sensitivity to Edges	Strong	Weak	Pooling A

**Table 6:** Salt-and-pepper noise profiles used in experiments to ensure reproducibility.

Noise Density (Amount)	Description	Example in Python 3.13.5 (skimage.util.random_noise)
0.10 (10%)	Light corruption; ~1 in 10 samples replaced	random_noise(x, mode = ‘s&p’, amount = 0.10)
0.20 (20%)	Moderate corruption; affects local features	random_noise(x, mode = ‘s&p’, amount = 0.20)
0.30 (30%)	Heavy corruption; severe loss of fine detail	random_noise(x, mode = ‘s&p’, amount = 0.30)

**Table 7:** Signal deviation metrics under 20% noise for different pooling methods (lower is better).

Noise Type	Pooling	MD	STD	MSE	1 − *ρ*
Salt-and-Pepper	Max Pooling	0.16	0.07	0.05	0.23
	Average Pooling	0.02	0.05	0.01	0.18
	Pooling A	0.02	0.07	0.01	0.11
	Pooling B	0.09	0.15	0.02	0.27
Gaussian	Max Pooling	0.41	0.01	0.22	0.70
	Average Pooling	0.01	0.00	0.02	0.33
	Pooling A	0.02	0.03	0.02	0.15
	Pooling B	0.29	0.02	0.09	0.12
Speckle	Max Pooling	0.17	0.03	0.05	0.16
	Average Pooling	0.02	0.08	0.01	0.06
	Pooling A	0.03	0.08	0.02	0.08
	Pooling B	0.09	0.13	0.04	0.10
Mixture Noise	Max Pooling	0.27	0.06	0.11	0.44
	Average Pooling	0.04	0.06	0.01	0.20
	Pooling A	0.02	0.05	0.02	0.20
	Pooling B	0.16	0.06	0.03	0.20
No Noise	Max Pooling	0.14	0.07	0.05	0.18
	Average Pooling	0.02	0.06	0.01	0.13
	Pooling A	0.03	0.08	0.01	0.09
	Pooling B	0.09	0.16	0.03	0.27

**Table 8:** Noise robustness evaluation summarization.

Noise Type	Best Metric (Per Pooling)	Worst Performing
Salt-and-Pepper	Pooling A shows the lowest MSE and highest correlation; Average Pooling is competitive.	Max Pooling
Gaussian	Pooling A achieves best 1 − *ρ*; Average Pooling slightly better in MD, STD, and MSE; Pooling B performs closely to A.	Max Pooling
Speckle	Average Pooling performs best across all metrics; Pooling A remains a close second.	Max Pooling
Mixture Noise	Pooling A achieves better 1 − *ρ*; Average Pooling slightly better in MSE and MD.	Max Pooling
No Noise	Pooling A shows best 1 − *ρ*; Average Pooling marginally better in MSE and STD.	Max Pooling

**Table 9:** Class-wise performance under clean and Gaussian noise conditions.

True Class	Predicted (Clean)—Top Confusion	Predicted (Gaussian Noise σ = 0.2)—Top Confusion	Comment
Walking	96% Walking (4% Jogging)	80% Walking (17% Jogging, 3% Standing)	Misclassified mainly as Jogging under noise
Jogging	96% Jogging (4% Walking)	78% Jogging (19% Walking, 3% Upstairs)	Overlaps with Walking under noise
Sitting	98% Sitting (2% Standing)	97% Sitting (3% Standing)	Robust to Gaussian noise
Standing	98% Standing (2% Sitting)	95% Standing (5% Sitting)	Slight degradation
Upstairs	96% Upstairs (4% Downstairs)	81% Upstairs (14% Downstairs, 5% Jogging)	Degraded, confuses with Downstairs
Downstairs	94% Downstairs (6% Upstairs)	82% Downstairs (16% Upstairs, 2% Jogging)	Degraded, confuses with Upstairs

**Table 10:** Latency and computational cost of pooling strategies for edge deployment (1D CNN).

Pooling Type	Latency per Window (ms)	FLOPs Estimate	Epochs to Converge	Epochs to Converge	Total Training Time (min)	Total Training Time (min)	Model Size (MB)	Model Size (MB)
			1D CNN	Two-Dimensional CNN	1D CNN	Two-Dimensional CNN	1D CNN	Two-Dimensional CNN
Average Pooling	0.05	12.2	50	46	35	45	1.33	3.01
Max Pooling	0.06	13.3	43	45	28	33	1.21	2.88
Pooling A	0.08	13.1	45	39	30	39	1.33	3.12
Pooling B	0.15	14.1	48	41	37	48	1.45	3.98

**Table 11:** Precision, recall, and F1 score per class for histogram-based CNN + FC models.

Metric/Method	Downstairs	Jogging	Sitting	Standing	Upstairs	Walking	Weighted Avg
Precision							
Avg Pooling	0.81	0.98	0.97	0.98	0.88	0.95	0.94
Proposed Pooling A	0.88	0.99	0.97	1.00	0.87	0.96	0.96
Proposed Pooling B	0.87	0.98	0.99	0.97	0.80	0.95	0.94
Recall							
Avg Pooling	0.78	0.98	0.97	0.98	0.88	0.95	0.93
Proposed Pooling A	0.88	0.99	0.97	1.00	0.87	0.96	0.96
Proposed Pooling B	0.87	0.98	0.99	0.97	0.80	0.95	0.94
F1 Score							
Avg Pooling	0.79	0.98	0.97	0.98	0.88	0.95	0.93
Proposed Pooling A	0.88	0.99	0.97	1.00	0.87	0.96	0.96
Proposed Pooling B	0.87	0.98	0.99	0.97	0.80	0.95	0.94

**Table 12:** Quantitative and qualitative evaluation of various pooling techniques.

Metric/Class	Proposed Pooling A	Proposed Pooling B	Average Pooling	Preferred Approach
Accuracy (Weighted Avg)	96.5%	93.3%	93%	Pooling A (highest accuracy)
F1 Score (Downstairs)	0.89	0.85	0.79	Pooling A
F1 Score (Upstairs)	0.87	0.80	0.88	Average Pooling (slightly higher F1)
Recall (Upstairs)	0.87	0.80	0.88	Average Pooling
Precision (Upstairs)	0.87	0.80	0.88	Average Pooling
F1 Score (Jogging)	0.99	0.97	0.97	Pooling A (highest)
F1 Score (Sitting)	0.97	0.99	0.96	Pooling B (slightly better)
F1 Score (Standing)	1.00	0.96	0.97	Pooling A
F1 Score (Walking)	0.96	0.94	0.94	Pooling A
ROC (weighted Average)	0.98	0.93	0.94	Pooling A
Computational Cost	Moderate	Moderate	Low	Average Pooling
Sensitivity to Noise	Low	Moderate	Low	Pooling A/Avg
Sensitivity to Edges	Strong	Moderate	Weak	Pooling A

**Table 13:** Effect of histogram bin size.

Input Representation	Bin Size	Accuracy (%)	Macro F1	Weighted F1	Remarks
Histogram Encoding	50	93.8	0.93	0.94	Coarse bins, lower resolution
Histogram Encoding	100	95.0	0.95	0.95	Balanced trade-off
Histogram Encoding	200	95.1	0.95	0.95	Slight gain, higher cost
Raw Window (2D CNN)	–	92.6	0.92	0.92	Transformation ablated

**Table 14:** Class-wise performance on overlapping sliding windows (stride = 50% of window size).

Activity	Precision	Recall	F1 Score	Comparison vs. Non-Overlapping (20%)
Downstairs	0.82	0.80	0.81	−5
Jogging	0.96	0.95	0.95	−1
Sitting	0.97	0.97	0.97	−1
Standing	0.98	0.97	0.97	−3
Upstairs	0.88	0.85	0.86	−8
Walking	0.96	0.95	0.95	−4
Macro Avg	0.93	0.91	0.92	−4
Weighted Avg	0.94	0.92	0.93	−3

**Table 15:** False positive rates under motion artifact perturbations.

Activity (True Label)	Noise-Free Accuracy (%)	With Motion Artifact (Avg. Shaking Amplitude 0.5 g)	False Positive Rate (%)	Most Frequent Misclassification
Standing	99.8	92.1	7.9	Walking
Sitting	98.9	90.7	8.2	Standing
Walking	96.7	91.4	5.3	Jogging
Jogging	95.2	91.9	3.3	Walking
Upstairs	93.5	86.4	7.1	Downstairs
Downstairs	92.7	85.5	7.2	Upstairs

**Table 16:** Per-class accuracy (%) across models and pooling strategies.

	Histogram-Aware CNN + FC (Average Pooling)	Histogram-Aware CNN + FC (Proposed Pooling A)	Histogram-Aware CNN + FC (Proposed Pooling B)	One-Dimensional CNN (Average Pooling)	One-Dimensional CNN (Pooling A)
Downstairs	78.3	87.8	87.3	77.8	85.8
Jogging	98.2	98.9	98.1	97.3	95.6
Sitting	97.0	97.4	98.7	95.0	96.9
Standing	98.1	100	97.0	97.9	99.4
upstairs	88.3	86.5	80.0	86.3	95.8
Walking	95.0	96.1	95.4	94.3	97.3
weighted Average	94.0	96.5	94.3	93.0	95.1

**Table 17:** Component-wise contribution analysis (ablation study).

Model Variant	Histogram Encoding	ECP	CMV	Accuracy (%)
Baseline CNN	×	×	×	79.91
+Histogram Only	✓	×	×	91.6
+ECP Only	×	✓	×	87.4
+CMV Only	×	×	✓	83.6
+ECP + CMV	×	✓	✓	89.1
Full Model (ECP + CMV + Histogram)	✓	✓	✓	96.84
ECP + Histogram	✓	✓	×	96.5

**Table 18:** A qualitative comparison of the proposed method with several existing approaches.

Study	Dataset Used	Model Type	Feature Representation	Pooling Strategy	Noise Robustness	Reported Accuracy (%)	Remarks
Kwapisz et al. (2011) [ACM SigKDD] [1]	Original WISDM v1.0	Multilayer Perceptron (MLP)	Handcrafted features (mean, std, etc.)	N/A	Not addressed	91.7	Classical benchmark; no deep learning used
Walse et al. (2016) [ICTCS] [2]	WISDM v1.0	k-NN, J48, Random Forest	Manual statistical features	N/A	Not addressed	84–89	Focus on classical ML classifiers
Min et al. (2020) [IJC] [3]	WISDM v1.0	SVM, RF, KNN	Time-domain features	N/A	Not addressed	90.2 (best)	No deep architectures; limited generalization
Seelwal & Srinivas (2023) [JOEE] [4]	WISDM v1.1	CNN	Raw time-series input	Max pooling	Not addressed	92.8	Used basic CNN without preprocessing enhancements
Heydarian & Doyle (2023) [arXiv] [5]	rWISDM (Repaired WISDM)	CNN-LSTM Hybrid	Denoised signals	Max pooling	Basic denoising applied	93.1	Enhanced input via preprocessing, but no custom pooling
Sharen et al. (2024) [ESWA] [6]	WISDM v1.1	WISNet (Custom DNN)	Raw signals + domain features	Avg/Max pooling	Minimal robustness	94.5	Deep model, but no redundancy modeling or pooling innovation
Abdellatef et al. (2025) [Sci. Reports] [7]	WISDM v1.1	Multi-layer CNN	Raw time-series	Avg pooling	Not addressed	94.2	Strong architecture but lacks input transformations
This Work (2025)	Based on WISDM	Two-dimensional CNN + Histogram	Raw time-series + Histogram-to-RGB image	Proposed Pooling A & B	Robust to Gaussian, S&P, Mixed noise	96.5	First to combine redundancy modeling + pooling design for noise-aware HAR

**Table 19:** Cross-dataset results (proposed vs. baselines).

Dataset	Model/Pooling	Accuracy 2D Histogram	Accuracy 1D Histogram	Macro F1	Notes
WISDM	Proposed (Pooling A)	96.5	95.1	0.95	Our main benchmark, segmentation-based windows
UCI-HAR	Proposed (Pooling A)	95.2	93.0	0.94	Slightly below SOTA (>97%), confirms transferability
MobiAct	Proposed (Pooling A)	94.7	93.1	0.93	Generalizes but drops in activities with high inter-user variability
WISDM	Average Pooling (CNN)	94.1	93.0	0.92	Baseline comparison

## Data Availability

The WISDM dataset used in this study is publicly available from the official WISDM repository and can be freely accessed for research purposes.
